# Graphite Web: web tool for gene set analysis exploiting pathway topology

**DOI:** 10.1093/nar/gkt386

**Published:** 2013-05-10

**Authors:** Gabriele Sales, Enrica Calura, Paolo Martini, Chiara Romualdi

**Affiliations:** Department of Biology, University of Padova, Via U. Bassi 58/B, 35121 Padova, Italy

## Abstract

Graphite web is a novel web tool for pathway analyses and network visualization for gene expression data of both microarray and RNA-seq experiments. Several pathway analyses have been proposed either in the univariate or in the global and multivariate context to tackle the complexity and the interpretation of expression results. These methods can be further divided into ‘topological’ and ‘non-topological’ methods according to their ability to gain power from pathway topology. Biological pathways are, in fact, not only gene lists but can be represented through a network where genes and connections are, respectively, nodes and edges. To this day, the most used approaches are non-topological and univariate although they miss the relationship among genes. On the contrary, topological and multivariate approaches are more powerful, but difficult to be used by researchers without bioinformatic skills. Here we present Graphite web, the first public web server for pathway analysis on gene expression data that combines topological and multivariate pathway analyses with an efficient system of interactive network visualizations for easy results interpretation. Specifically, Graphite web implements five different gene set analyses on three model organisms and two pathway databases. Graphite Web is freely available at http://graphiteweb.bio.unipd.it/.

## INTRODUCTION

The complexity of the regulatory mechanisms of the cell combined with the difficulties in the interpretation of high-throughput ‘omic’ data has led to the development of a myriad of novel computational methods for data management, analysis and integration.

Two approaches can be recognized: (i) those based on unsupervised approaches where gene expression data is combined with protein–protein and protein–DNA interactions networks to the identification of modules or subnetworks of the entire network (1–4) and (ii) those based on supervised approaches where predefined gene sets are tested to evaluate their involvement in a specific experimental condition (5–15). The focus of this work is on this second type of approaches.

The aim of the supervised methods is to identify sets of genes with coordinated expression and/or concentration changes in different biological conditions, unravelling the complexity of cellular regulatory processes. In this context, the use of pathways as gene sets is of great help in simplifying the complexity and the interpretation of gene expression measurements.

Gene set analyses can be subdivided into the classical enrichment analysis, working on gene lists selected through a gene-level test, and the novel global and multivariate approaches that define a model for the whole gene set.

In general, these two approaches have two fundamentally different null hypotheses. The first type hypothesizes that a given gene set has the same level of association with a phenotype as the rest of the genes. The second type only considers the genes within a gene set and hypothesizes that there is no gene in the gene set associated with the phenotype (6). These two approaches have been termed ‘competitive’ and ‘self-contained’, respectively (16). It is worth noting that multivariate approaches can be competitive or self-contained, and conversely, competitive can be multivariate.

The main drawbacks with ‘competitive’ methods are (i) the assumption that genes are independent along with (ii) the use of a cut-off threshold for the selection of differentially expressed genes (DEGs). In this way, many genes with moderate but meaningful expression changes are discarded by the strict cut-off value, which leads to a reduction in statistical power. On the other hand, global and multivariate approaches relax the assumption of independence among genes of the same gene set and identify possibly moderate, but coordinated, expression/concentration changes that cannot be detected by the previous approaches without depending on any arbitrary cut-offs.

A biological pathway is not a mere list of genes but represents the biologic relations between the macromolecules within a cell. They can be represented through graphs where genes and their relations are, respectively, nodes and edges. As a result, pathway analyses can be further divided into ‘topological’ and ‘non-topological’ depending on their ability to gain power from the information stored in the graph. Nearly all gene set analyses consider pathways as a simple gene list, ignoring the topological information. The reason for this is 2-fold: (i) the difficulty of retrieving the information of pathway topology and converting it to a gene network, (ii) the difficulty of including graph topology within statistical models.

Pathway annotation comprises chemical compounds mediating interactions and different types of gene groups (e.g. protein complexes or gene families) that are usually represented as single nodes but whose measures are not available using microarray or RNA-seq data. It is therefore necessary to convert pathways from their native format to gene-only networks. Our group has recently developed ‘graphite’ (17), a Bioconductor package that taking the information from different databases, interprets pathway formats and reconstruct the correspondent gene–gene networks following specific biologically driven rules. ‘graphite’ (17) gives the unprecedented possibility to use pathway topology for gene expression data analysis. To address the issue of considering graph topologies within statistical models, we recently proposed a totally new method for topological pathway analysis, called CliPPER (10,18). CliPPER is a two-step empirical approach based on Gaussian graphical models, which identifies pathways with means or covariance matrices significantly different between experimental conditions. It also selects the portions of the pathway, called signal paths, which are associated the most with the given phenotype.

To this day, the most used approaches remain based on non-topological and univariate methods. These approaches completely miss the relationship among genes (6–9 among others) but offer an intuitive result interpretation. Topological and multivariate approaches are on the contrary more powerful, but are also difficult to use by researchers without bioinformatic skills.

Here we present Graphite web, the first public server for topological-based pathway analysis based on high-throughput gene expression data analyses. Graphite web combines topological and multivariate pathway analyses with an efficient and interactive system of network visualizations that allows an easy results interpretation. Specifically, Graphite web deals with microarray or RNA-seq data. It implements different multivariate gene set analyses [classical hypergeometric enrichment, global test (7), gene set enrichment analysis (GSEA) (6), signalling pathway impact analysis (SPIA) (5), CliPPER (10) on three model organisms (human, mouse and drosophila] and two pathway databases [KEGG (19) and Reactome (20)]. The implementation of different types of analysis will open up to the user the significant possibility to directly benchmark the performances of different algorithms on her data.

## WEB TOOL IMPLEMENTATION

Graphite web has two sections: (i) mapping and interactive browsing of pathway networks and (ii) pathway analysis using gene expression data from either microarray or sequencing technology.

Before giving the details of these two separate sections, we briefly introduce the way ‘graphite’ converts pathway topology into gene-only networks.

### Pathway topology conversion, visualization and web implementation

Pathway annotations comprise a myriad of interactions, reactions and regulations, which are often too rich to be represented in a network. Challenges are posed in particular by the presence of chemical compounds mediating interactions and by different types of gene groups (e.g. protein complexes or gene families) that are usually represented as single nodes.

The core of Graphite web is ‘graphite’ (17), a Bioconductor tool recently developed by our group for the storage, interpretation and conversion of pathway topology to gene-only networks. ‘graphite’ discriminates between different types of biological gene groups and propagates gene connections through chemical compounds. Specifically, protein complexes are expanded into a clique (all proteins connected to the others), while the gene families are expanded without connections among them; see (17) for more details.

Chemical compounds are not usually measured with high-throughput technologies; however, pathway annotations contain several compound-mediated interactions (interactions for which a compound acts as a bridge between two elements). As the trivial elimination of the compounds strongly bias the topology, ‘graphite’ takes into account cell compartment membership and propagates the signal connecting the compound-mediated elements [see (17) for more details].

Graphite web uses the gene-only networks derived from this conversion for the topological analyses and for the result visualization.

Tissue specificity is a critical point to better comprehend and interpret the final results; an imprecise model affects the efficacy of the analyses. Unfortunately, current pathway databases represent pathways regardless of the cell type and tissue they occur in. Thus, the user has to be aware that the topology provided by Graphite web represents the integration of the information available in different experimental conditions.

Apart from ‘graphite’, Graphite web uses other Bioconductor packages for pathway analysis (SPIA, sigPathway, globaltest, goseq, clipper), identification of DEGs (edgeR, limma), imputation of missing values (impute) among others. Graphite web will be automatically updated every 6 months whenever the new Bioconductor release will be published. Cytoscape web (21) is used to provide an interactive view of the networks.

### Pathways browsing

The Browse section allows the user to visualize genes (nodes) on pathways (networks), using a colour scale proportional to the fold changes of the genes (if they are provided by the user). Gene IDs in the input (Gene IDs supported EntrezGene, Ensemble gene ID, HUGO Symbol), are automatically converted to EntrezGenes and mapped on all the pathways of the selected database. The following steps are required:

Step 1: Select the organism.
Step 2: Select the database.
Step 3: Upload the input file or paste the input in a text box.

Input files can be tab-delimited with two columns: the first column is the gene ID and the second column is the log fold change (optional) associated to the gene. In case only the expression directions are available (over/under expression), the user can associate to the genes the values −1 and +1.

The results are divided in sections reporting, respectively, (i) the table of all the pathways with at least one mapped gene, and for each pathway (ii) the interactive network-based visualization with nodes coloured according to the fold change provided.

### Analysis

A brief overview of the gene set approaches implemented in Graphite web is reported below. According to the statistic used, each method is categorized as competitive or self-contained and topological or non-topological. For an extensive review and critical discussion see (16,22–27).

#### Enrichment analysis (competitive and non-topological)

Enrichment analysis is based on Fisher Exact test and estimates the chance probability of observing a given number of genes of a particular pathway among the selected DEGs. For each pathway, a two-way contingency table is generated as follows:

**Table:** 

	DEG	EEG	tot
∈ G	n_G,deg_	n_G,eeg_	N_G_
∉ G	n_GC,deg_	n_GC,eeg_	N_GC_
tot	N_deg_	N_eeg_	N

where EEG means equally expressed genes, N is the total number of genes screened, G is the pathway and GC is the complement of G. N_i_ and n_i_ are the frequencies of genes belonging to each table cell. Then, the probability P of observing at least n_G,deg_ genes of a functional category within a group of N_deg_ genes is given by: 
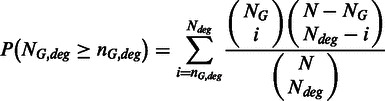



Then, Ps are adjusted using Benjamini and Hochberg method (28).

In case of RNA-seq count data, the statistical test widely used to identify DEGs is based on the negative binomial distribution (accounting for a quadratic dependence between mean and variance) (29). In this case, the read count defines the power of the test and given the strict dependence between read count and gene length, longer genes are characterized by a higher statistical power to be detected as differentially expressed. It has been shown that these differences in length/power if not properly assessed can introduce some bias in the final results (30). Graphite web allows an optional accounting for this bias using the *P*-value correction for the gene length as implemented in ‘goseq’ Bioconductor package.

#### Global test for group of genes (self-contained and non-topological)

Global test is based on a penalized logistic regression model. The general idea is to find the genes within a pathway whose combination of expression profiles best predicts clinical data (subdivision in samples classes). In this model, the dependent variable is the vector of classes, while the covariates are the expression profiles of the genes belonging to the pathway. The model has a total number of parameters equal to the number of genes in the pathways. Typically, using gene expression data from high-throughput technology, the number of samples is much lower than the number of genes within a pathway. This type of model is defined non-identifiable, as we do not have a sufficient number of replicate (samples) to estimate the parameters. To cope with this unbalance structure of the data, Goeman *et al.* (7) proposed a penalized regression model where the coefficient of some genes are shrinked toward zero, reducing the number of parameters to be estimated.

#### Gene set enrichment analysis (self-contained or competitive and non-topological)

GSEA was originally proposed by (8,9). The procedure is based on the following steps: (i) select a statistic to compare groups of samples (e.g. *t*-test), (ii) rank the entire list of genes according to the value of this statistic, (iii) define a pathway G, and compare the distribution of the statistic of G and GC.

In the original version GSEA, a weighted Kolmogorov–Smirnov (K–S) test was proposed for the comparison between G and GC test distributions, where the gene weights were given by the absolute value of the statistic. The significance of weighted K–S test was estimated through a permutational approach. The authors suggest permuting samples if the sample size is sufficiently large and to permute the genes otherwise. A normalization strategy was also proposed for the K–S statistic to take into account the pathway dimension. In these years, several improvements have been described using alternative ranking metrics, enrichment statistics and several variations of the significance estimation schemes; see (6,31–33) among others. Specifically, it was shown (6) that the differences in the correlation structure of each pathway could lead to a biased comparison among gene sets unless a normalization procedure is applied. To cope with this problem, Tian *et al.* (2005) proposed the use of the standard statistical approach for comparing mean shift of the G and the GC distributions: a one-sample *z*-test with a permutational approach. In particular, they described two different approaches: permutation of samples and permutation of genes. The first one leads to a self-contained test, the second one to a competitive test.

Given *t_i_*, the statistic of the gene *i* with *i* = 1 … *N* where *N* is the total number of genes, the two gene set statistics proposed by Tian *et al.* (2005) are as follows: 
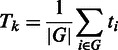

with its null distributions generated by permuting {*t_1_*, … , *t_N_*}; 
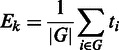

with its null distributions generated by permuting samples {*z_1_*, … , *z_p_*}.

It is important to note that although the formula for *E_k_* is the same as that of *T_k_*, their probability interpretations and hence their testing procedures are different. In *T_k_*, *t_i_* is deterministic and the gene set structure is random; in *E_k_*, the opposite is true (6).

After a proper standardization, we obtain *NT_k_* and *NE_k_* statistics. The correlation structure in gene sets can still give false positives for *NT_k_*; conversely, the gene set size can influence *NE_k_*. Then Tian *et al.* (2005) thus suggests taking as good candidate’s pathways that are significant for both *NT_k_* and *NE_k_*.

Graphite web implements Tian *et al*. (2005) GSEA statistics.

#### Signalling pathway impact analysis (competitive and topological)

The method proposed by Tarca *et al.* (2009) (5,34) calculates a score through the combination of several aspects of the data: the fold change of the DEGs, the pathway enrichment score and the topology of signalling pathways. Specifically, from a topological point of view, SPIA enhances the impact of a pathway if the DEGs tend to lie near the entry points of a pathway (gene upstream of the pathway).

SPIA needs as input the list of DEGs with their log fold changes and the complete list of gene names in the platform. SPIA then computes (i) the hypergeometric enrichment *P*-values, *pNDE*, (ii) a perturbation factor as a linear function of the perturbation factors of all genes in a given pathway, whose significance is calculated through a bootstrap approach, *pPERT*, and (iii) the combination of the two independent *P*-values (*pNDE* and *pPERT*), called *pG*. *pGs* are then adjusted for multiple testing using the false discovery rate (FDR) algorithm (35).

Each pathway is finally marked as activated (positive perturbation score = positively perturbed) or the inhibited (or negatively perturbed) (5).

#### Pathway analysis through Gaussian Graphical Models (CliPPER) (self-contained and topological)

Pathway dimensions are highly heterogeneous and we expect, from a biological point of view, that only some portions of a pathway would be involved, especially for large pathways. Among topological methods, however, none tries to identify the signal paths that are involved the most in the biological problem.

In this perspective, our group has developed CliPPER (10,18), a totally new approach to fill this gap. Specifically, CliPPER is an empirical method based on Gaussian graphical models that (i) selects pathways with covariance matrices or means significantly different between experimental conditions; and (ii) on such pathways, identifies the portions of a pathway, called signal paths, associated the most with the phenotype.

Different experimental conditions are usually compared in terms of their gene expression mean differences. However, the difference in mean expression levels does not necessarily result in a change of the interaction strength among genes. For example, a proportional increase of the expression of the genes A and B in one of two conditions will result in significantly different mean expression between the two conditions. The correlation strength between A and B, however, does not change. In this case, we would have pathways with significant altered mean expression levels but unaltered biological interactions.

If, on the contrary, transcripts abundances ratios are altered, we expect a significant alteration not only of their mean expression levels, but also of the strength of their connections. That corresponds to a change in the biological activity that can be captured by the measuring the expression variance.

CliPPER therefore searches for pathways strongly involved in a biological process by requesting that the mean or the variance of the expression levels result significantly altered between two conditions.

### Input files and data processing

**Figure 1.**
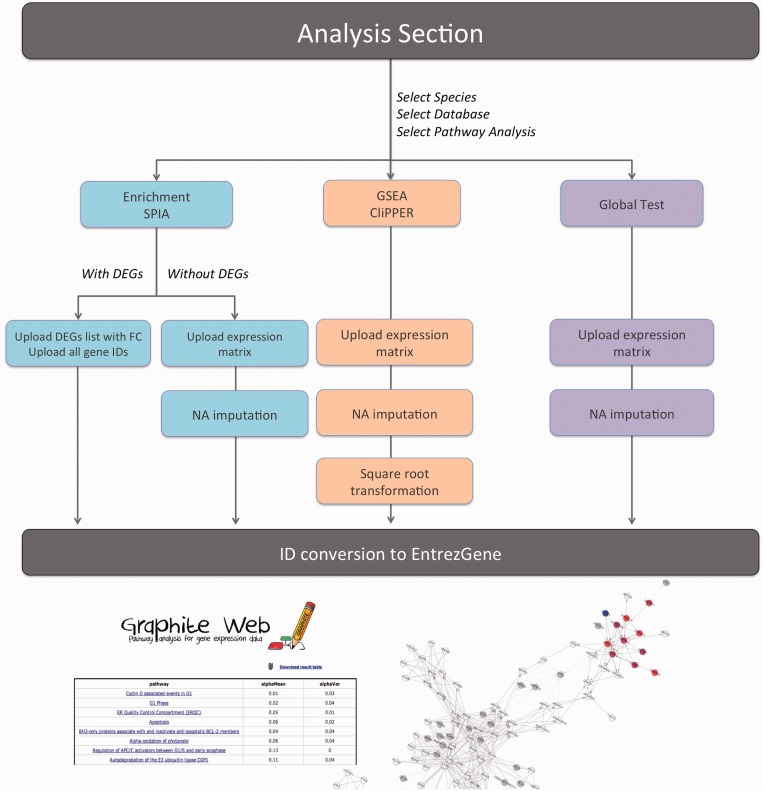
Schematic representation of Graphite web data processing according to the different analyses provided.

After selecting the type of analysis, the organism, the database and the type of data (microarray or RNA-seq), the user has (i) to choose the threshold for the minimum number of mapped gene within a pathway required for the pathway to be processed, (ii) to optionally insert the email for results notification and finally (iii) to upload input files. A scheme of the different data processes required by the analysis is shown in Figure 1.

Graphite Web takes as input tab-delimited files. Depending on the analysis selected and the user setting, these files can be matrices (samples on the columns and genes on the rows) or lists of DEGs.

Graphite web supports different types of IDs (EntrezGene, Ensemble gene ID and gene Symbol). Before the analysis begins, gene IDs are all converted, if necessary, to EntrezGene.

Enrichment analysis and SPIA require two separate files (i) the list of DEGs with fold change and (ii) the whole list of genes. Graphite web gives two possibilities: (i) the separate upload of these files, when the user has already performed differential expression, or (ii) the upload of the normalized expression matrix with the automatic detection of DEGs using an empirical Bayes test [(28), for microarray data as implemented in ‘limma’ Bioconductor package] or negative binomial test [(29), for RNA-seq data as implemented in ‘edgeR’ Bioconductor package].

Global test, GSEA and CliPPER require a normalized expression matrix.

Expression matrices should be tab-delimited text files where the first row should contain sample names (the sample name represents the sample class) and the first column the gene IDs. In case of missing values (represented by the ‘NA’ string) Graphite web automatically performs an imputation using k-nearest neighbor algorithm as implemented in ‘impute’ Bioconductor package. When the input data derives from an RNA-seq experiment, GSEA and CliPPER analyses are run over the square root transformed count data. It has been shown, indeed, that the square root of a Poisson-distributed variable is approximately normally distributed (36). Global test does not require any data transformations.

### Output and network visualization

Graphite web removes from the analysis all the pathways with a number of mapped genes (intersection of genes in the expression matrix and genes in the pathway) less than the user-defined threshold and generates a table reporting the names of at least 50 top-scoring pathways. For each pathway, an interactive graph represents the gene-only network. Genes are colour-coded according to their contribution to the analysis. Grey nodes are genes not available in the platform/matrix uploaded; they are nodes that did not contribute to the analysis. Coloured nodes (with colour proportional to the log fold change) correspond to genes differentially expressed (for hypergeometric and SPIA analysis) or that strongly contribute to the significance of the pathway (for globaltest, GSEA and clipper). White nodes are those genes not differentially expressed (for hypergeometric and SPIA) or that contribute little to the significance of the pathway (for globaltest, GSEA and clipper).

All the results can be downloaded (as a single zip file) and every analysis is stored at specific URL that can be accessed for 6 months from different IPs.

For each significant pathway, Graphite web allows the download of (i) the PDF image of the network with nodes colour-coded according to their contribution to the analysis; (ii) a text file with the list of genes (EntrezGene, Symbol, Description and score) belonging to the pathway and used for the analysis; (iii) a text file with the list of genes (EntrezGene, Symbol, Description) belonging to the pathway, but not used for the analysis (not present in the list of genes provided by the user) (iv) a SIF file to load the pathway network in an external software such as Cytoscape (37).

## CASE STUDIES

### The cancer genome atlas colorectal cancer data

As a benchmark case study, we used the gene expression data available from the TCGA project on colorectal cancer (CRC). Clinical information and normalized expression profiles on 220 individuals were downloaded from https://tcga-data.nci.nih.gov/docs/publications/coadread_2012/.

CRC ranks the third and second among all commonly encountered malignancies in terms of incidence and mortality, respectively. The high mortality rate of advanced CRC can be attributed to limited treatment options. In this perspective, the stage of a cancer is one of the most important factors in determining prognosis and treatment options. Stage codification is based on how far the cancer has grown into the wall of the intestine, whether it has reached nearby structures and whether it has spread to the lymph nodes or distant organs. It is usually quoted as I, II, III, IV, where a higher number indicates a more advanced cancer and likely a worse outcome. CRC stage I indicates that cancer has begun to spread, but is still in the inner lining; stage II indicates that cancer has spread to other organs near the colon or rectum but it has not reached lymph nodes; stage III indicates that cancer has spread to lymph nodes, but has not been carried to distant parts of the body, while stage IV indicates metastasis. Given the importance of the comprehension of the mechanisms that lead to the spread of the cancer on distant organs, in this example we focus on the transition between stage II and III. We select stage II and stage III from the whole cohort of patients obtaining a list of 137 individuals (82 of stage II and 55 of stage III). We then performed all the analyses provided by Graphite web.

It is worth noting that no DEGs have been identified using empirical Bayes test (38) (FDR ≤ 0.1). Our example demonstrates that in such situations, high-level pathway analyses are a valuable alternative to detect moderate but coordinate expression/concentration alterations. Given the absence of DEGs we proceed only with global test, GSEA and CliPPER.

Global test does not return significant pathways (FDR < 0.05).

GSEA identified (44) significant Reactome pathways with *NTk* statistic (gene permutation strategy) and no pathways with *NEk* statistic (sample permutation). In the home page of Graphite web, the complete lists of the significant pathways with the adjusted *P*-value for KEGG and Reactome databases are available.

Mechanism of CCR has not been fully characterized yet; however, it has been known that deregulation of cell cycle and apoptosis contribute to cancer progression and both this pathways are present in the analysis reported (39,40). In addition, almost all the top significant pathways are immune related. It has been shown that the balancing between the activation and the suppression of the host immune system against CCR play a key role determining the cancer progression (41). In particular, we find pathways involving NFKB and JAK-STAT signalling, Toll-like receptors (TLRs) signalling, especially the MyD88-dependent cascades, and interleukins signalling pathways. It has been demonstrated that TLRs signalling directly promote and support intestinal carcinogenesis, and in fact a reduced expression of TLR4 is associated with tumour growth and metastatic status (42). Finally the involvement of MyD88-dependent TLRs signalling in tumour growth and progression has been demonstrated both in mice model (43) and in CRC cell lines (44). On the other hand, inhibition of JAK1, 2/STAT3 signalling induces apoptosis, cell cycle arrest and reduces tumour cell invasion in CRC cells (45). IL-6 is a multi-functional pro-inflammatory cytokine that has crucial roles in tumour progression through growth-promotion, anti-apoptotic activity and modulation of immune function, and thus is a strong candidate for mediating both local and systemic cancer-associated inflammatory responses. It is of interest, therefore, that the IL-6/JAK/STAT pathway has emerged as a key player in cancer-associated inflammation (46).

Exploiting the possibility offered by Graphite web to investigate the most influential genes within each pathway, we uncover a series of genes that perfectly corroborate previous observations. In particular, we find NFKB2, NFKBIA, JAK2, IL6, FOS, TLR4, PIK3CB, TNF, STAT3, STAT5, S100A12, IRAK3.

**Figure 2.**
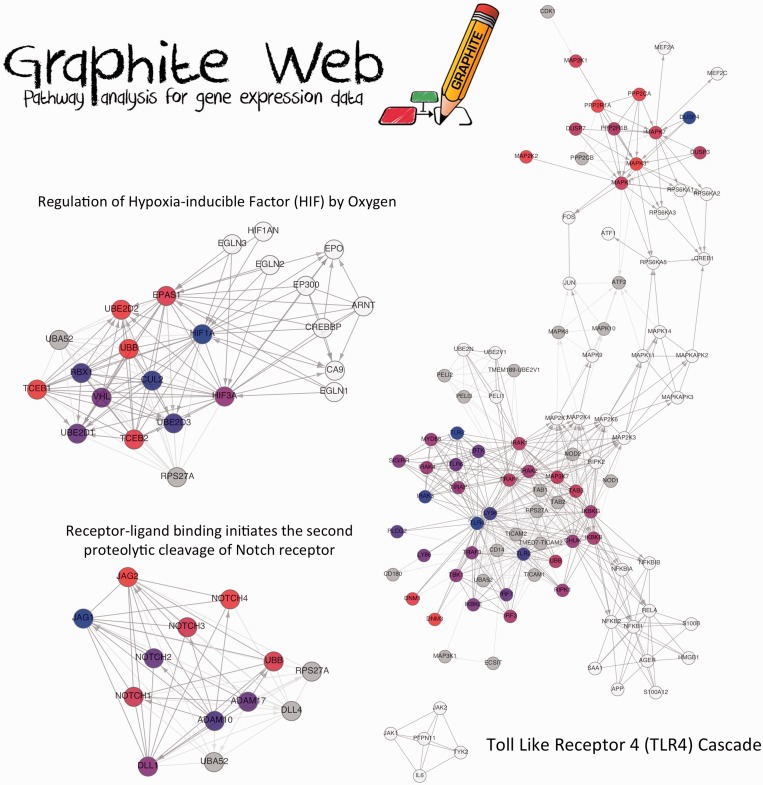
A selection of CliPPER results on CRC data set. Three significant pathways are shown. Coloured nodes represent the portions of the pathways mostly involved in the progression from stage II to stage III identified by CliPPER. The colour of the nodes is proportional to their fold change (stage III versus stage II).

Although CliPPER is based on a different null hypothesis with respect to GSEA, their results partly overlap (see the home page of Graphite web for the complete list of results). The statistical significance of this overlap for Reactome and KEGG results has been estimated using hypergeometric distribution (*P* = 0.0003 for KEGG and *P* = 0.001 for Reactome). CliPPER identifies 80 pathways (Reactome with means or concentration matrices significant altered in the two classes). Many of them are associated to TLRs (specifically TLR4), apoptosis, cell cycles, but also NOTCH, Wnt and Hypoxia-inducible Factor signalling pathways. They are all known to have key roles in CRC. Figure 2 shows three of the most interesting pathways identified by CliPPER, where coloured nodes represent the portion of the pathway involved the most in the pathology, and the colours themselves are proportional to the gene fold change (stage III versus stage II). Notch signalling is an important molecular pathway involved in the determination of cell fate. In recent years, this signalling has been frequently reported to play a critical role in maintaining progenitor/stem cell population as well as a balance between cell proliferation, differentiation and apoptosis (47). Notch signalling is often and aberrantly activated by hypoxia during tumour progression. Specifically, the activation of Jagged2 by hypoxia in tumour cells induced epithelial to mesenchymal transition and it also promoted cell survival *in vitro*, playing a critical role in tumour progression and metastasis (Figure 2) (48). Many of the adaptations to hypoxia are mediated by the activation of specific genes through hypoxia-inducible factor (HIF) such as HIF1 and HIF2 (also known as EPAS1). Their key regulatory subunits, HIF-1α and HIF-2α, are induced similarly by hypoxia, but their functional roles in cancer may be distinct and isoform specific. Xenograft studies revealed that HIF-1α deficiency inhibited overall tumour growth, whereas deficiency of HIF-2α stimulated tumour growth (Figure 2) (49).

As a practical example of CliPPER demonstrating its usefulness during the interpretation of results, we investigate in detail the TLR4 pathway that is known to be involved in CRC.

CliPPER highlights two TLR4-mediated signalling pathways that correspond to two-coloured path of the network. The first one is the MAPK area with (MAPK1, MAPK3, MAPK7, some subunits of protein phosphatase 2 and some dual specificity phosphatase, DUSP). The ERK/MAPK pathway is one of the most important for cell proliferation, and its overexpression and activation are commonly detected in CRC. Several evidence indicates that overexpression and activation of ERK MAPK play an important part in the progression of this cancer (50). The second one is the TRL4 area (with MYOD88, TRL3, IRAKs and TRAFs genes) that, as reported before, is perfectly coherent with CRC progression.

While a complete investigation of biological relevance of all the results reported by Graphite web is beyond the scope of this work, these results highlight the usefulness and power of the tool even in cases where biological groups are highly similar and classical inferential approaches fail to provide new insights.

## CONCLUSIONS

Pathway analysis aims at identifying groups of functionally related genes that show coordinated expression/concentration changes. Recently, pathway analyses moved from algorithms using mere gene lists to new ones exploiting the topology that define gene connections. Unfortunate limits to the use of these new methods are (i) the availability of the pathways as gene networks in which nodes are only genes, (ii) a user-friendly access to topological statistical analysis usually implemented in the R language. Graphite web has been developed to face both issues. The core of Graphite web is graphite (17), a tool developed by our group for the storage, interpretation and conversion of pathway topology to gene-only networks using biological-driven rules. Graphite web implements a totally new system of pathway visualization and provides an easy access to multivariate and topological pathway analyses. The combination of a pathway-specific visualization with powerful gene set analyses gives to the user the possibility to explore in great detail signalling pathways and the position of the influential genes within them.

## FUNDING

Funding for open access charge: University of Padova [CPDA119031 to C.R.].

*Conflict of interest statement.* None declared.
